# Loss of *wwox* expression in zebrafish embryos causes edema and alters Ca^2+^ dynamics

**DOI:** 10.7717/peerj.727

**Published:** 2015-01-27

**Authors:** Yusuke Tsuruwaka, Masataka Konishi, Eriko Shimada

**Affiliations:** 1 Marine Bioresource Exploration Research Group, Japan Agency for Marine-Earth Science and Technology (JAMSTEC), Yokosuka, Japan; 2 School of Materials Science, Japan Advanced Institute of Science and Technology (JAIST), Nomi, Ishikawa, Japan; 3 Department of Animal Science, University of California, Davis, CA, USA

**Keywords:** Zebrafish, Intracellular calcium, Yellow cameleon, *WWOX*

## Abstract

We investigated the role of the WW domain-containing oxidoreductase (*wwox*) gene in the embryonic development of zebrafish, with particular emphasis on intracellular Ca^2+^ dynamics because Ca^2+^ is an important intracellular messenger. Comparisons between zebrafish *wwox* and human *WWOX* sequences identified highly conserved domain structures. *wwox* was expressed in developing heart tissues in the zebrafish embryo. Moreover, *wwox* knockdown induced pericardial edema with similarities to conditions observed in human breast cancer. The *wwox* knockdown embryos with the edema died within a week. High Ca^2+^ levels were observed at the boundary between the edema and yolk in *wwox* knockdown embryos.

## Introduction

WW domain-containing oxidoreductase (*WWOX*) is a tumor suppressor gene that is reportedly absent in several cancers, including those of breast, prostate, and ovary (Lewandowska et al., 2009; Salah, Aqeilan & Huebner, 2010), and has been associated with cancer progression in *Wwox* conditional knockout (KO) mice (Yang & Zhang, 2008; Gardenswartz & Aqeilan, 2014; Li et al., 2014). *WWOX* is a 46-kDa protein with two N-terminal WW domains and a central short-chain dehydrogenase/reductase domain (SDR domain). These WW domains may be involved in protein–protein interactions, whereas the SDR domain may play roles in steroid metabolism and bone development (Salah et al., 2013; Li et al., 2014). *WWOX* is essential for vertebrate development and bone metabolism, which are dependent on intracellular Ca^2+^ levels (Aqeilan et al., 2008). Accordingly, Ca^2+^ signaling is known to be a key messenger in various biological phenomena (Niki et al., 1996; Slusarski & Pelegri, 2007), and intracellular Ca^2+^ levels are reportedly increased in advanced sarcoma (Britschgi et al., 2013). However, it is difficult to monitor intracellular Ca^2+^ levels in various developmental stages of mammals because their embryos are not transparent. Hence, we focused on zebrafish (*Danio rerio*), which provides an established model to study human cancer and offers several advantages over mammalian model systems, including rapid development of transparent embryos (Amatruda et al., 2002; Feitsma & Cuppen, 2008). In the present study, we investigated the function of *wwox* in vertebrate development using *D. rerio* as a model organism, and assessed the ensuing effects on intracellular Ca^2+^ dynamics.

## Materials and Methods

### Materials

Adult wild-type zebrafish were purchased from a local pet store (Remix, Nagoya, Japan) and were maintained at 28.5 °C in 2-L tank equipped with a system that circulates conditioned filtered tap water under a 14-h/10-h light/dark cycle. Embryos were generated from natural crosses. Fertilized eggs were rinsed with autoclaved 1/3 Ringer’s solution containing 1.67 mM HEPES, 38.7 mM NaCl, 0.97 mM potassium chloride, and 0.60 mM calcium chloride (pH 7.2) to remove debris. Constructs of yellow cameleon 2.12 (pCS2-YC2.12) were kindly provided by A. Miyawaki (RIKEN Advanced Science Institute, Wako, Japan). The open reading frame of YC2.12 was inserted between *Bam*HI and *Eco*RI restriction sites located downstream of the SP6 promoter sequence in the pCS2 vector. Using SP6 RNA polymerase from the mMessage mMachine SP6 *in vitro* transcription kit (Life Technologies, Carlsbad, CA, USA), 5′-capped YC2.12 mRNA was synthesized *in vitro* using pCS2-YC2.12 as a template. Synthesized mRNA from a single expected electrophoretic band was purified, dissolved in nuclease-free water, and stored at −80  °C before use. No approval was required to conduct studies on fish according to the Ministry of Education, Culture, Sports, Science and Technology, Notice No. 71 (in effect since June 1, 2006).

### Total RNA extraction

Total RNA was extracted from whole fish embryos at various developmental stages. After rinsing with 10 mM phosphate-buffered saline (PBS) at pH 7.4 (2.9 mM NaH_2_PO_4_, 9.0 mM Na_2_HPO_4_, and 138 mM NaCl) and 100% ethanol three times, total RNA was extracted using an SV Total RNA Isolation System (Promega Corp., Madison, WI, USA) according to the manufacturer’s instructions.

### Polymerase chain reaction (PCR) and complementary DNA (cDNA) cloning

Target *wwox* cDNA was synthesized from total RNA (2.7 µg) using a SMART RACE cDNA Amplification Kit (Takara Bio, Shiga, Japan) and was amplified using an Advantage 2 PCR Kit (Takara Bio) with the following primers (accession no. BC044560): forward GCTGCCAAACAAACACCTC and reverse TTCCCAGATAACTTCCGAGC. The PCR program comprised an initial denaturation step at 94 °C for 2 min, followed by 35 cycles of 94 °C for 15 s, 55 °C for 30 s, 72 °C for 1 min, and a final extension step at 72 °C for 7 min. PCR products were purified by agarose gel electrophoresis, and PCR products were cloned using a pTAC-2 vector containing M13F and M13R, a DynaExpress TA PCR Cloning Kit (pTAC-2), and Jet Competent Cells (Biodynamics Laboratory, Tokyo, Japan). The M13 universal primer (forward GTAAAACGACGGCCAGT) was used in sequencing analyses with GENETYX ver. 8.0 software (Genetyx, Tokyo, Japan), the Clustal W multiple sequence alignment algorithm (www.clustal.org/), and the Basic Local Alignment Search Tool (http://blast.ncbi.nlm.nih.gov/Blast.cgi), and homology between zebrafish *wwox* and human *WWOX* cDNA sequences was calculated. RNA rescue experiments were performed after preparing *wwox* mRNA (ADH domain region) by replacing pTAC-2 with the pCS-2 vector. The open reading frame of the ADH domain was inserted between *Bam*HI and *XhoI* restriction sites located downstream of the SP6 promoter sequence in the pCS2 vector. Relative mRNA abundance was then semiquantitated after scanning gels, and pixel intensities were determined for each band and were normalized to those of *β*-actin using ImageJ software (National Institutes of Health, Bethesda, Washington, D.C. USA). Zebrafish *β*-actin (accession no. AF057040.1) forward and reverse primers were TGGCATTGCTGACCGTATGC and GTCATGGACGCCCATTGTGA, respectively. Experiments were repeated six times, and data are presented as the mean ± standard error of the mean (SEM).

### Construction of antisense morpholinos (MOs) and small interfering RNA (siRNA)

MOs (Gene Tools, LLC, Philomath, OR, USA) and siRNA (B-Bridge International, Inc., Tokyo, Japan) were used to knockdown the expression of *wwox* (accession number: BC044560). In these experiments, a *wwox* MO was designed to disrupt *wwox* translation, and *wwox* siRNA was designed to target the N-terminal *wwox* WW domain sequence. MO sequences were as follows: Danio-*wwox* MO, 5′-TATTTGAGAGCCGCCATTGCGAAAT-3′ (3′-fluorescein), and 5-mis-Danio *wwox* MO (negative control), 5′-TAaTTGAcAGCgGCgATTGCcAAAT-3′ (3′-fluorescein). The standard MO control 5′-CCTCTTACCTCAGTTACAATTTATA-3′ (3′-fluorescein) was obtained from Gene Tools and was used as a positive control. The *wwox* siRNA sequence was 5′-GAGCAAAGCCCGUGUGGAA-3′. Approximately 3 nL of MOs (1 mM) or siRNA (50 µM) were injected into single-cell zebrafish embryos, and phenotype rescue was achieved by co-injection of a target gene RNA (ADH domain) that does not bind MO or siRNA, as previously described in ZFIN (https://wiki.zfin.org/display/prot/RNAi+for+Zebrafish) and Bill et al. (2009).

### Microinjection

Microinjection needles were prepared from model GD-1 microcapillaries using a model PC-10 micropipette puller (Narishige, Tokyo, Japan). Embryos were placed in an agarose chamber and were soaked in 1/3 Ringer’s solution. Approximately 3 nL of synthetic mRNA (0.5 ng/mL) and siRNA (50 µM) were then injected into blastodiscs of each single-cell embryo. Subsequently, embryos were transferred to new dishes that contained the same medium and were incubated at 28.5 °C. Numbers of eggs were 2460 in *wwox* MO, 2590 in *wwox* siRNA, and 770 in rescue experiments. Positive yellow fluorescent protein (YFP) expression was detected in mRNA-injected embryos using a Leica L2 microscope equipped with a L5 FL Stereofluorescence System (Leica, Tokyo, Japan). Images were captured using a CoolSNAP cooled 5.0 charge-coupled device camera with RS Image Software (Roper Industries, Inc., Sarasota, FL, USA). Embryos were embedded in 3% methylcellulose (Sigma-Aldrich, St. Louis, MO, USA) to straighten their bodies, and body length images were measured from the head to the tail along with body axis in lateral view (*n* = 30) in three independent experiments using RS Image Software. Eye sizes were measured and the longest diameters of eyeballs were recorded. Data are presented as means ± SEM, and differences between groups were identified using Student’s *t*-test and were considered significant when *p* < 0.01.

### Ca^2+^ imaging

Embryos expressing YC2.12 mRNA were mounted in glass-bottom (0.08 mm) Petri dishes (35 mm; Matsunami Glass, Ind., Ltd., Osaka, Japan) that contained 1/3 Ringer’s solution supplemented with 3% methylcellulose. YC2.12 is a Förster resonance energy transfer-based indicator that exploits conformational changes following binding of calcium to the CaM motif, which alters the distance between the relative orientations of cyan fluorescent protein and YFP, and is reflected by energy transfer between the markers. Fluorescence was quantified as previously described (Miyawaki et al., 1999; Tsuruwaka et al., 2007). Embryos at prim-15 stage (30 h post fertiliation; hpf) were analyzed because Ca^2+^ imaging could not be performed after 33 hpf due to the remarkable pigment patterns on zebrafish bodies (Tsuruwaka et al., 2007).

### Oligonucleotide tailing with digoxigenin (DIG)-dUTP/dATP

The *wwox* antisense probe 5′-TAAATGGACACTTGCGGGAATACAAACACT-3′ was obtained from Hokkaido System Science Co., Ltd. (Sapporo, Japan), and was labeled with DIG-11-UTP using a DIG Oligonucleotide Tailing Kit, 2nd Generation (Roche, Mannheim, Germany). Probe concentrations were estimated using an absorption spectrometer.

### *In situ* hybridization

In these experiments, embryos were manually dechorionated, fixed overnight in 4% buffered paraformaldehyde (PFA) at 4 °C, dehydrated with a series of graded methanol (MeOH) solutions, and stored at −20 °C in MeOH for further use. After thawing, embryos were immersed in a series of 75%, 50%, and 25% MeOH solutions in PBS containing 0.1% Tween 20 (PBST; pH 7.0) for 5 min at room temperature (RT). Endogenous peroxidase activity was inhibited by incubation in 3% H_2_O_2_ in MeOH for 20 min at RT. Embryos were then rehydrated using a graded MeOH series to 100% PBST, and were permeabilized using 10 µg/mL proteinase K (Promega Corp.) in PBST at 37 °C for 30 min. To stop the reaction, embryos were washed with 2 mg/mL glycine in PBST for 2 min, refixed in 4% buffered PFA for 20 min, and then washed five times in PBST for 5 min each time. Subsequently, embryos were prehybridized for 1–8 h at 55 °C in hybridization buffer containing 50% formamide, 5 × saline–sodium citrate (SSC; 20 × SSC, 3 M NaCl, and 300 mM trisodium citrate), 0.1% Tween 20, 500 µg/mL tRNA (Sigma-Aldrich), and 50 µg/mL heparin (Sigma–Aldrich; pH 6.0–6.5). Before hybridization, the probe was heated for 10 min at 80 °C. Hybridization was performed overnight at 55 °C in 100 µL of hybridization buffer containing 50–100 ng of the DIG-labeled oligonucleotide probe.

After hybridization, embryos were briefly washed in hybridization buffer (without tRNA and heparin), and were then washed in a gradient of hybridization buffer/2 × SSC of 75%/25%, 50%/50%, and 25%/75% at 55 °C for 30 min each, with a final wash in 100% 2 × SSC. Subsequently, embryos were washed two times in 2 × SSC for 30 min at RT, followed by a 10-min wash at RT over a gradient of 0.2 × SSC/PBST of 75%/25%, 50%/50%, and 25%/75%, and a final wash in 100% PBST. Embryos were then preabsorbed for 2 h at RT under slow agitation in antibody buffer comprising PBST, 2% sheep serum, and 2 mg/mL bovine serum albumin. Subsequently, embryos were incubated with Anti-Digoxigenin-Fluorescein Fab fragments (dilution, 1:1000; Roche) in antibody buffer overnight at 4 °C under gentle agitation according to the manufacturer’s protocol.

After antibody incubation, embryos were washed six times for 15 min each in PBST, and microscope analyses were performed on embryos mounted in glass-bottom dishes using a Zeiss Axiovert 200 fluorescence microscope (Carl Zeiss, Oberkochen, Germany).

## Results

### Homology between zebrafish *wwox* and human *WWOX* sequences

**Figure 1:**
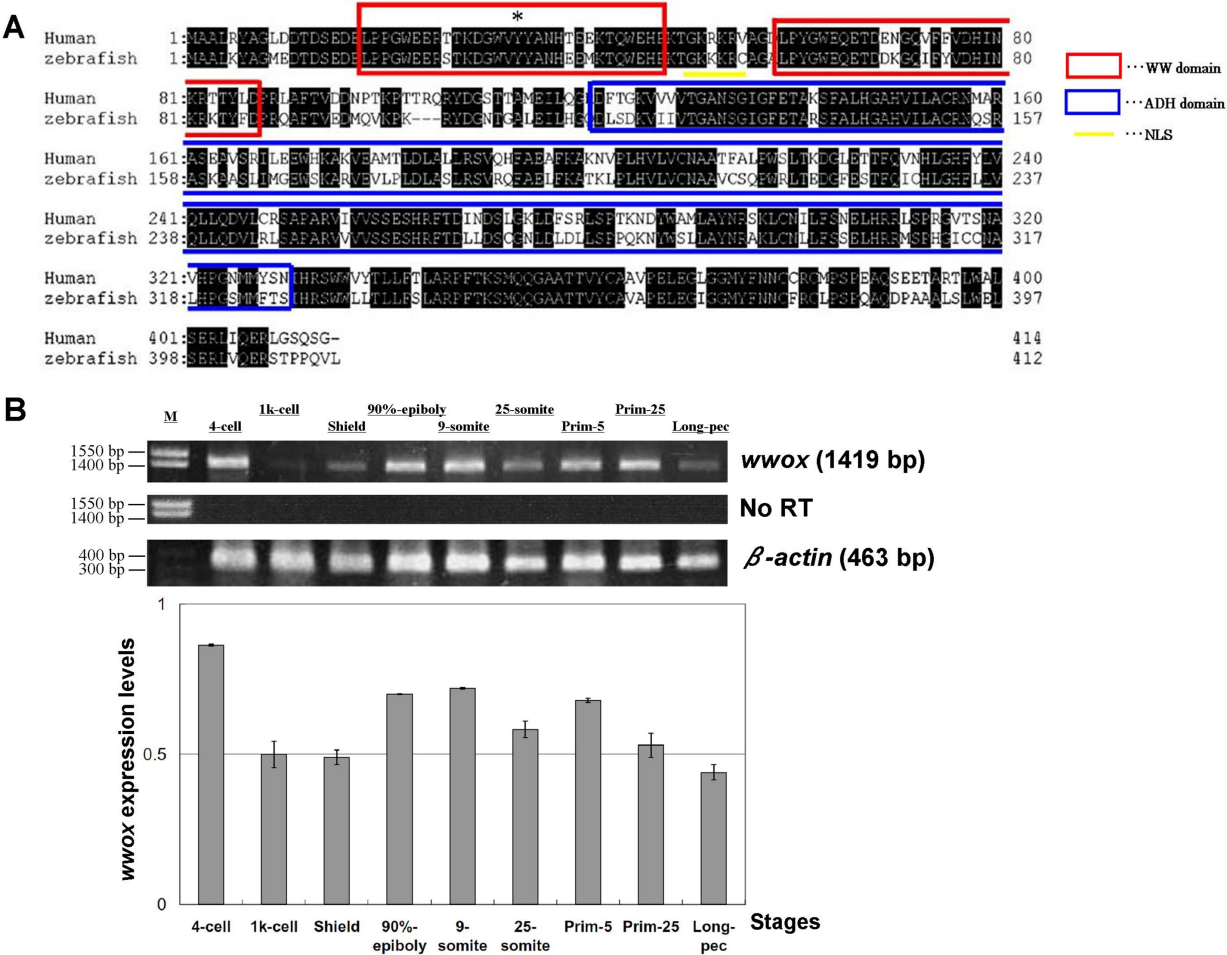
Alignment of human and zebrafish *WWOX* amino acid sequences, and expression of zebrafish *wwox* using RT-PCR. (A) Human and zebrafish *WWOX* amino acid sequence alignments Human *WWOX* (accession no. AF187014), zebrafish *wwox* (accession no. BC044560), the WW domain (amino acids 18–47 and 59–87, circled with red boxes), the NLS (amino acids 50–55, underlined), the ADH domain (amino acids 121–330, circled with a blue box) and Tyr^33^ (*) are shown. (B) Expression of zebrafish *wwox* at various developmental stages was determined using RT-PCR and gel image analyses were normalized to *β*-actin expression (accession no. AF057040.1). Data are presented as means ± SEM; M, marker; No RT, RT-PCR reaction without reverse transcriptase.

Homology of zebrafish and human *wwox* sequences was assessed using sequences retrieved from the GenBank database (http://www.ncbi.nlm.nih.gov/genbank/). Amino acid homology between these species was 72% (Fig. 1A), and WW and ADH domain structures and nuclear localization signals (NLS) were highly conserved. Tyr^33^ was also conserved in the zebrafish *wwox* (Fig. 1A), suggesting that Tyr^33^ phosphorylation may activate *WWOX* and lead to activation of p53 and JNK1 in zebrafish, as it does in humans (Chang et al., 2003). Subsequent RT-PCR analyses indicated that *wwox* was expressed at each embryonic developmental stage of zebrafish (Fig. 1B).

### Knockdown and detection of zebrafish *wwox*

**Figure 2:**
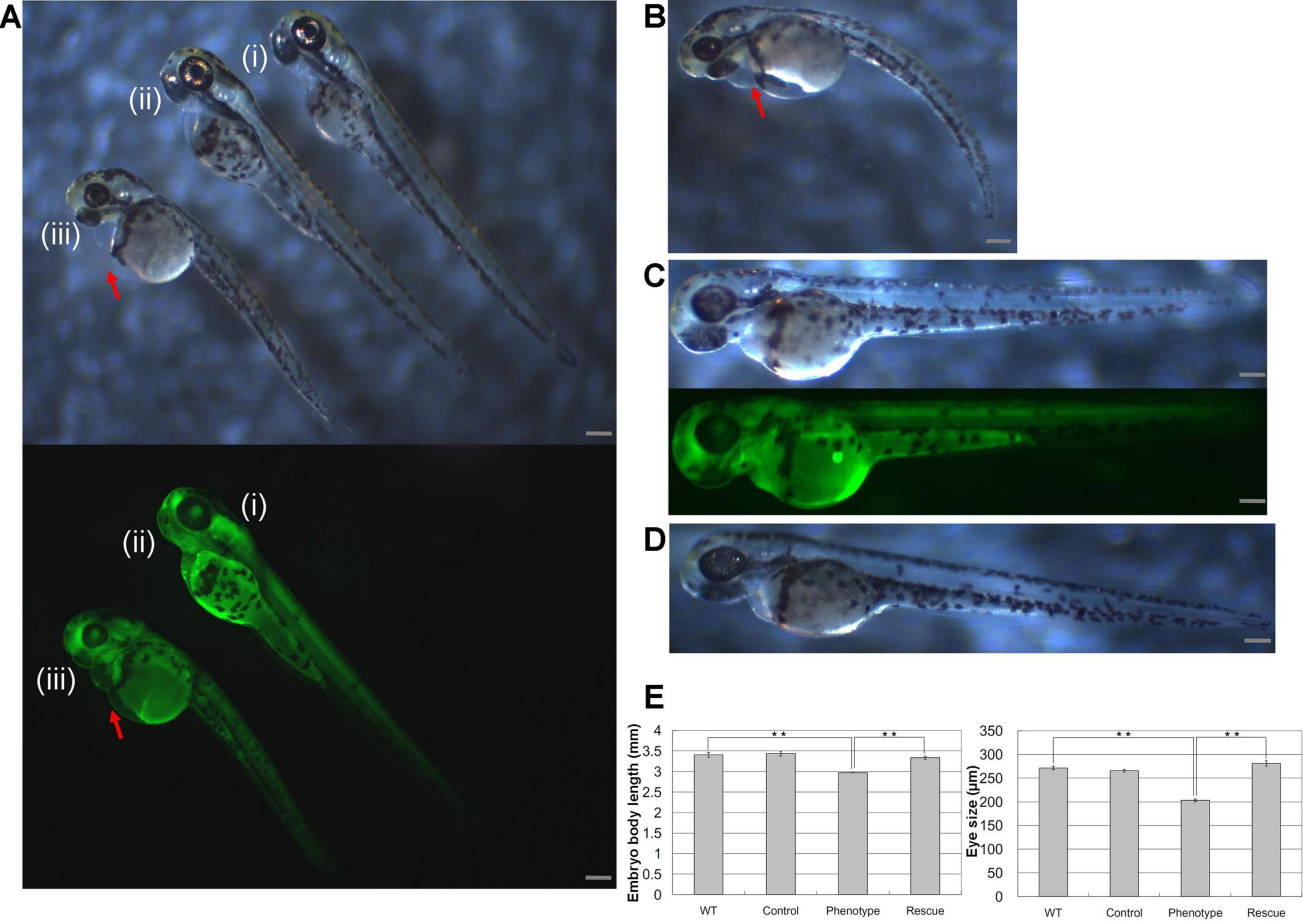
Knockdown of *wwox* induced pericardial edema and reduced eye formation in embryos during the long-pec stage at 48 h post fertilization. Arrows indicate edema; (A) Upper, A bright-field image of a (i) wild-type embryo; (ii) *wwox* negative-control MO-injected embryo; and (iii) *wwox* MO-injected embryo; Lower, fluorescence image of the upper panel. (B) A *wwox* siRNA-injected embryo. (C) A *wwox* rescued MO-injected embryo (upper, bright-field image; lower, fluorescence image). (D) A *wwox* rescued siRNA-injected embryo. (E) Quantification of body lengths and eye sizes of long-pec embryos; 30 embryos were examined in three independent experiments and data are expressed as means ± SEM (^∗∗^*p* < 0.01); WT, wildtype; Scale bars, 250 µm.

**Figure 3:**
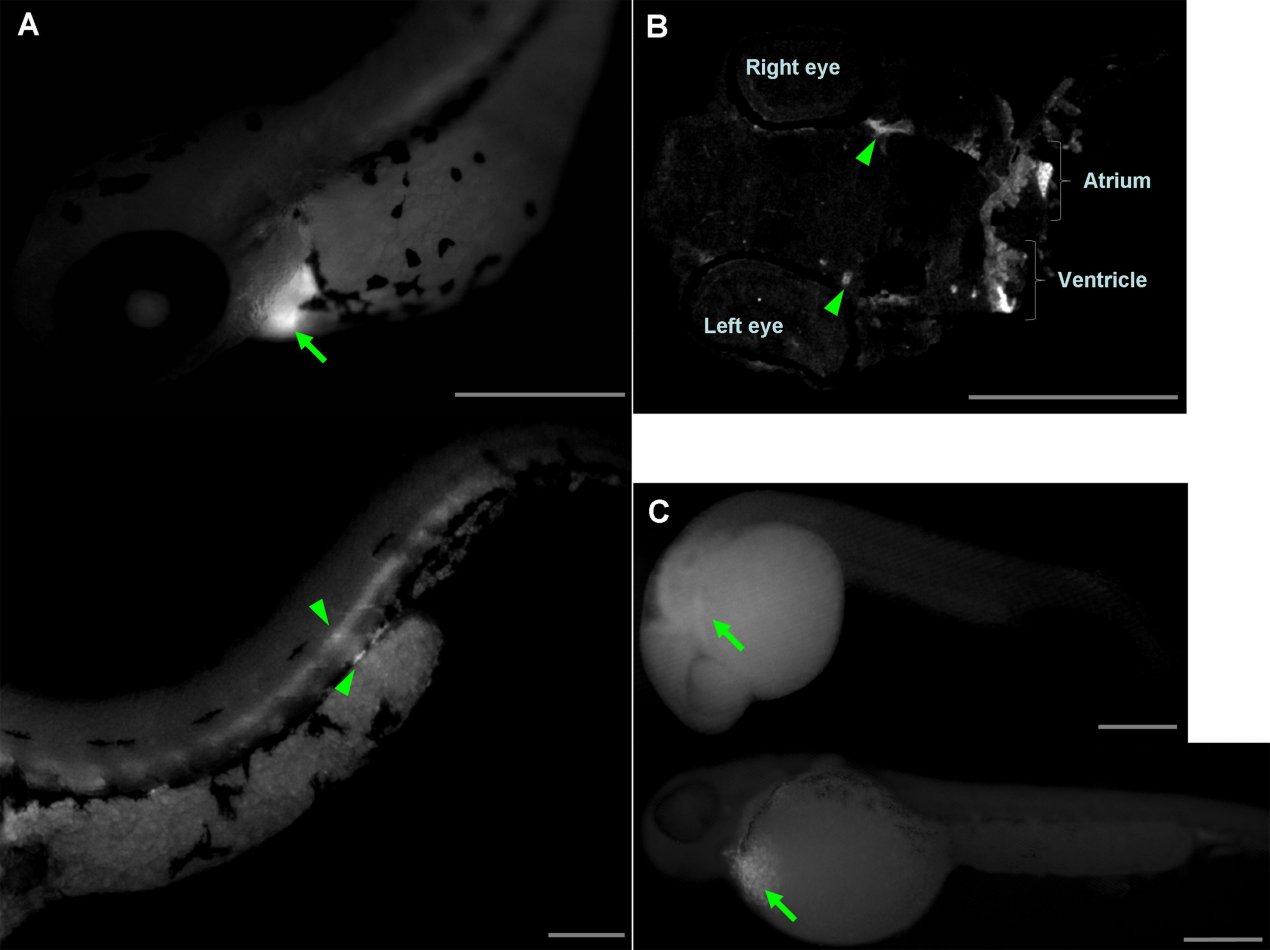
Determination of *wwox* expression in developing zebrafish hearts using fluorescent whole-mount *in situ* hybridization. (A) Lateral view of the heart (upper) and tail (lower) of the embryo at the long-pec stage; Arrow, ventricle; arrow heads, putative digestive organs. (B) A cardiac tissue section of the long-pec embryo; Arrow heads show optic nerves. (C) Lateral view of embryos at prim-5 (upper) and prim-25 (lower); Arrows indicate putative ventricles; Scale bars, 250 µm.

Injections of *wwox* targeting MOs into zebrafish embryos resulted in phenotypic alterations that were characteristic of pericardial edema at 48 hpf during the long-pec stage (Fig. 2A). A similar phenotype was produced by siRNA knockdown during development (Fig. 2B), with 56% and 62% penetrance of edema in 48 hpf embryos after injections with MOs and siRNA, respectively. Edema was particularly severe around the heart and caused death of embryos within 1 week. However, *wwox* mRNA rescued the phenotype in MO and siRNA injected embryos (Figs. 2C and 2D). Knockdown of *wwox* caused noticeable reductions in eye sizes and body lengths, and embryos had twisted backbones (Fig. 2E). The formation of edema was apparent from the segmentation period and a distinct phenotype was observed during the long-pec stage (data not shown). In subsequent fluorescence whole-mount *in situ* hybridization experiments, *wwox* expression was detected in developing hearts of zebrafish embryos, and was further visualized in tissue sections using *in situ* hybridization and immunocytochemical staining methods. Strong *wwox* expression was detected in developing hearts at 48 hpf and weak expression was observed in putative digestive organs (Fig. 3A). Ventricles had higher *wwox* expression than atriums, and weak localized expression was detected in optic nerves (Fig. 3B). Moreover, *wwox* expression became detectable from the prim-5 stage (24 hpf) and was clear at the prim-25 stage (36 hpf) during formation of the developing heart (Fig. 3C).

### Analysis of intracellular Ca^2+^ dynamics after edema formation

**Figure 4:**
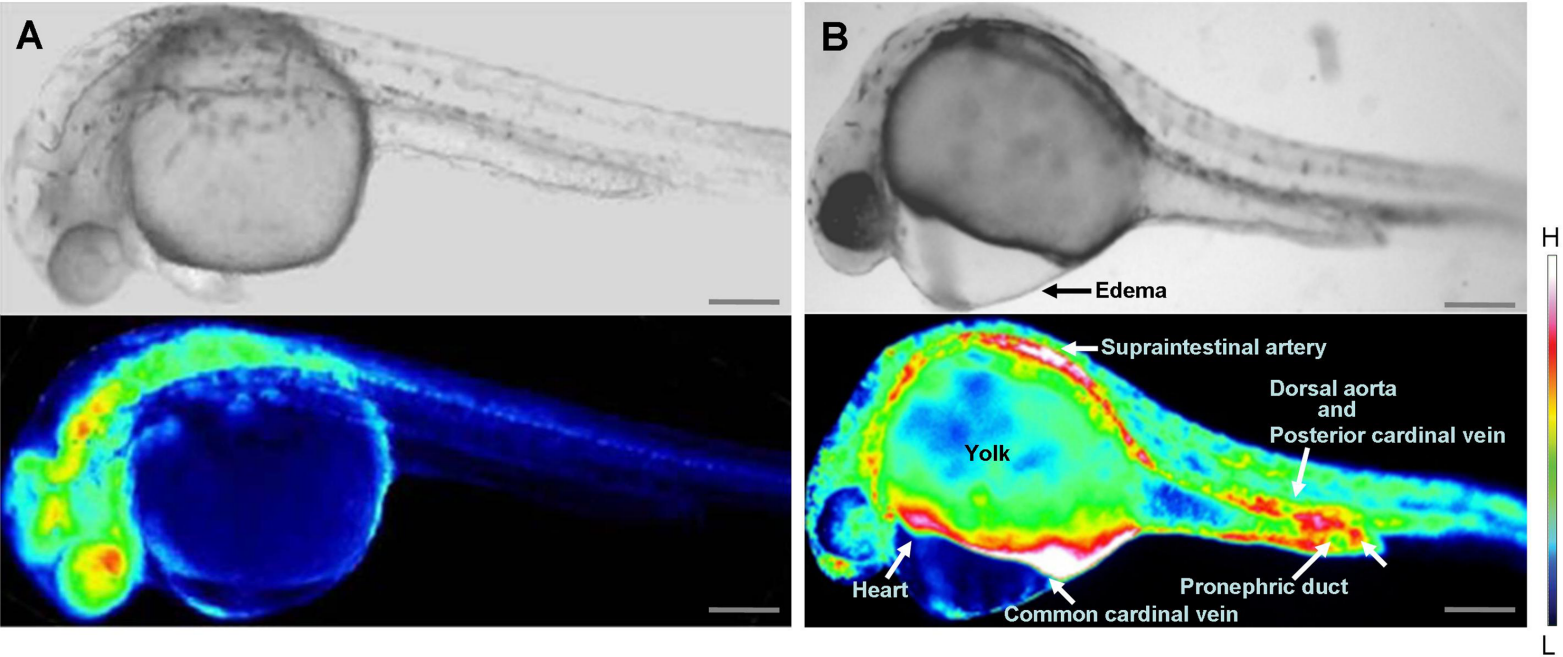
Comparison of intracellular Ca^2+^ dynamics in prim-15 stage (30 hpf) embryos. Upper, bright field image; lower, color-coded image; (A) wildtype; (B) *wwox* knockdown produced edema formation and induced altered Ca^2+^ dynamics; Scale bars, 250 µm. The color-coded image shows Ca^2+^ levels as white (high Ca^2+^) and blue (low Ca^2+^).

Inhibition of *wwox* expression caused morphological abnormalities, and Ca^2+^ signaling patterns are reportedly altered during zebrafish morphogenesis (Tsuruwaka et al., 2007; Webb & Miller, 2007). Therefore, we analyzed intracellular Ca^2+^ dynamics after edema formation in embryos that had been simultaneous transfected with yellow cameleon, YC2.12, and siRNA. Patterns of Ca^2+^ dynamics strikingly differed between *wwox* knockdown embryos and normal fish (Fig. 4), with maximal Ca^2+^ levels at the boundary between the edema and yolk.

Relatively high rates of intracellular Ca^2+^ dynamics were observed among areas of heart tissue and in putative digestive organs that expressed *wwox*. High Ca^2+^ levels were observed in putative digestive organs, but no corresponding morphological changes were noted.

## Discussion

Human *WWOX* has been functionally associated with breast cancers in humans, and zebrafish and human *WWOX* amino acid sequences are highly homologous. Thus, despite the absence of breast tissue in zebrafish, zebrafish and human *WWOX* may have similar functions. In the present study, knockdown of *wwox* in zebrafish embryos produced a pericardial edema phenotype.

Nunez, Ludes-Meyers & Aldaz (2006) reported high *WWOX* mRNA expression in human breast, ovaries, testes, and prostate tissues, and *WWOX* expression was reportedly absent in several breast cancers (Lewandowska et al., 2009). Although zebrafish do not possess mammary tissues such as those in humans, edema formation was observed around the pectoral region in *wwox* deficient zebrafish, as shown in mice (Qu et al., 2013). Previous studies implicate *WWOX* in the development of gastric carcinoma (Aqeilan et al., 2004), suggesting that additional effects of *wwox* dysregulation may be associated with the present zebrafish phenotype. Accordingly, high levels of Ca^2+^ were observed in tissues with high *wwox* expression, with the exception of the dorsal region, which was subject to deleterious digestive tract and blood vessel environments that reflected dysfunctions of the heart pump. Analyses of Ca^2+^ dynamics in *wwox* knockdown zebrafish suggested novel roles of dorsal–posterior regions other than the pericardial edema. Specifically, *wwox* knockdown embryos displayed early lethality that was similar to that in *Wwox* KO mice, which died by 4 weeks of age (Aqeilan et al., 2007). Other phenotypes observed in *Wwox* KO mice included abnormal bone structures and reduced postnatal growth (Ludes-Meyers et al., 2009). Developmental retardation, as indicated by small eyes and heads and abnormal bone formations, was also observed in *wwox* knockdown zebrafish embryos. Similarly, postnatal day 18 *Wwox* KO mice showed notable differences in bone structure, with significantly decreased bone volume and cortical thickness compared with wild-type mice. Moreover, a *Wwox* gene mutation (*lde*) was identified in rats and led to phenotypic lethal dwarfism with epilepsy (Suzuki et al., 2009). These defects in bone formation further suggest that *wwox* knockdown alters Ca^2+^ dynamics. In a previous study, Webb & Miller (2007) showed that Ca^2+^ signaling changes with somite formation during zebrafish development. Moreover, alterations of Ca^2+^ dynamics at various sites suggest that multiple organ failure have led to death in the present embryos.

In summary, we demonstrated the utility of the present fish model in studies of *wwox* gene function. The present data show strong effects of *wwox* knockdown in developing heart tissue and weaker effects on putative digestive organs. Subsequently, high Ca^2+^ levels were observed at ventral–dorsal–posterior regions along with the blood circulation of *wwox* knockdown zebrafish embryos. Therefore, inhibition of *wwox* could have pointed a non-cell-autonomous phenotype. These data suggest roles of *wwox* in multiple tissues during fish embryonic development, as previously reported in mice. In morphological studies, inhibition of *wwox* expression led to edema formation, resembling that previously shown in developing tumors (Jiang & Hui, 2008). Finally, abnormal Ca^2+^ dynamics were observed after inhibition of *wwox* expression, suggesting that monitoring of Ca^2+^ levels may provide an additional indicator for phenotype analyses. However, further studies are required to determine whether the present zebrafish edema was derived from carcinogenic processes.

## Conclusion

Zebrafish *wwox* was expressed highly in developing heart tissues and detectably in putative digestive organs. Loss of *wwox* function caused edema in the pectoral region, developmental retardations such as small eyes and short body lengths, and altered Ca^2+^ dynamics.

## Additional Information and Declarations

### DNA Deposition

The following information was supplied regarding the deposition of DNA sequences: GenBank accession no. BC044560.
